# Successful treatment of acute device thrombosis of patent foramen ovale with slow infusion of low-dose thrombolytic therapy

**DOI:** 10.1093/ehjcr/ytae360

**Published:** 2024-08-30

**Authors:** Selahattin Akyol, Emrah Bayam, Anıl Avci, Ünal Güler, Ramazan Kargin

**Affiliations:** Kartal Kosuyolu High Specialty Training and Research Hospital, Department of Cardiology, University of Medical Sciences, Istanbul, Turkey; Kartal Kosuyolu High Specialty Training and Research Hospital, Department of Cardiology, University of Medical Sciences, Istanbul, Turkey; Kartal Kosuyolu High Specialty Training and Research Hospital, Department of Cardiology, University of Medical Sciences, Istanbul, Turkey; Kartal Kosuyolu High Specialty Training and Research Hospital, Department of Cardiology, University of Medical Sciences, Istanbul, Turkey; Kartal Kosuyolu High Specialty Training and Research Hospital, Department of Cardiology, University of Medical Sciences, Istanbul, Turkey

**Keywords:** Case report, Patent foramen ovale, Occluder device thrombosis, Thrombolytic therapy

## Abstract

**Background:**

Percutaneous closure of patent foramen ovale (PFO) is used in selected individuals to eliminate the risk of recurrent cerebral embolism due to paradoxical embolization. Although device thrombosis is rare, it can cause serious complications. Herein, we report a 40-year-old woman who developed acute PFO closure device-associated thrombus and was subsequently treated with slow infusion of low-dose tissue plasminogen activator (t-PA) (25 mg/6 h).

**Case summary:**

A 40-year-old woman was admitted to the hospital because of an cerebrovascular accident (CVA). Computed tomography and magnetic resonance imaging of the brain demonstrated the presence of an ischaemic lesion in the right cerebellar infarct. Since no pathological finding was detected that could cause CVA, it was considered that there might be paradoxical embolism due to PFO. Percutaneous PFO closure was decided by the heart and brain team. The occluder was implanted under transoesophageal echocardiography (TEE) and fluoroscopy guidance. Although activated clotting time was 250 s, hypermobile acute thrombus measuring 11 × 5 mm was seen on the left atrial side of the PFO device. Slow infusion of low-dose t-PA treatment was given. As soon as after a single-dose t-PA, control TEE was performed and it was seen that almost the entire thrombus was lysed. The patient did not have any complications during the treatment period.

**Discussion:**

Acute PFO device thrombosis is a rare but important complication. If there is no contraindication for lytic treatment in acutely developing large PFO device thrombosis, slow infusion of low-dose t-PA may be useful.

**Learning points**

- Performing percutaneous patent foramen ovale (PFO) closure under transoesophageal echocardiography guidance is important in terms of evaluating the success, results, and complications of the procedure.
- Acute PFO device thrombosis is a rare but important complication. If there is no contraindication for lytic treatment in acutely developing large PFO device thrombosis, slow infusion of low-dose t-PA may be utilized.

## Introduction

Percutaneous closure of patent foramen ovale (PFO) is used in selected individuals to eliminate the risk of recurrent cerebral embolism due to paradoxical embolization. Although device thrombosis is rare, it can cause serious complications. Herein, we report a 40-year-old woman who developed acute PFO closure device-associated thrombus and was subsequently treated with slow infusion of low-dose tissue plasminogen activator (t-PA) (25 mg/6 h).

## Case presentation

A 40-year-old woman was admitted to the hospital due to cerebrovascular accident (CVA). She presented with transient vertigo, left-sided lower extremity paresis, and ataxia. Computed tomographic (CT) and magnetic resonance scan of the brain demonstrated the presence of an ischaemic lesion in the right cerebellum. Laboratory investigation did not reveal any abnormalities or thrombophilia. Chest radiography, electroencephalogram, electrocardiography, Holter monitoring, and carotid ultrasonography did not reveal any abnormal finding. Conditions that might be associated with hypercoagulation were investigated, and genetic tests were performed; the results were unremarkable. The neurologist recommended 75 mg clopidogrel and 100 mg acetylsalicylic for the management of CVA.

**Figure 1:**
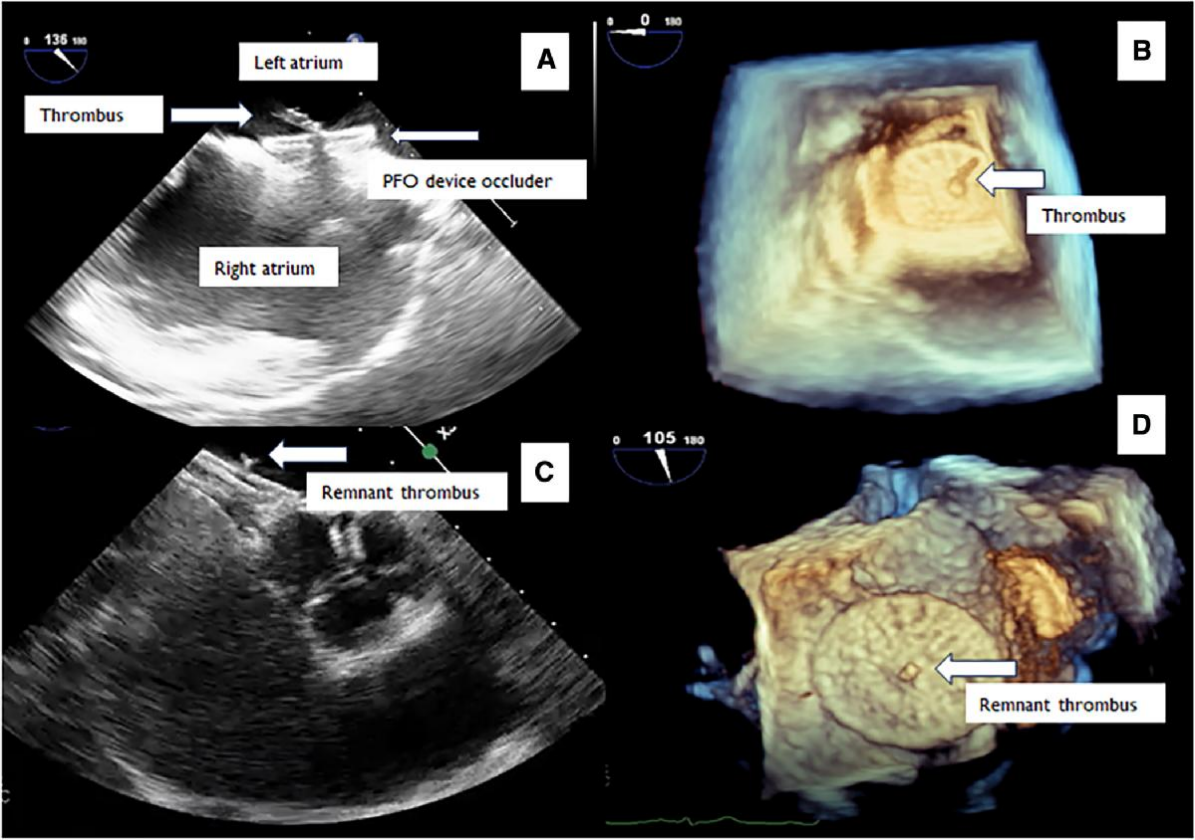
TEE images of PFO device before and after thrombolytic therapy.

The patient underwent transthoracic and transoesophageal echocardiography (TEE) for the assessment of any cardiac thromboembolism. The evaluation of atria, ventricles, valves, and left atrial appendage did not delineate any abnormal finding. The interatrial septum was hypermobile, and significant right-to-left shunt was observed through the PFO at rest without Valsalva manoeuvre (see Supplementary material online, *Video S1*). Since no pathological finding was detected that could cause CVA, it was considered that there might be paradoxical embolism due to PFO. Percutaneous PFO closure was decided by the heart and brain team. A 28/28 mm MemoPart PFO Occluder device (Lepu Medical) was implanted to close the PFO and prevent further possible paradoxical embolization. Due to the hypermobile interatrial septum, a larger device was used. The occluder was implanted under TEE and fluoroscopy guidance. After device implantation, the interatrial septum was immobilized and TEE did not reveal any residual shunt. One hundred units per kilogram bolus i.v. heparin was given through the right femoral vein. Activated clotting time (ACT) measurement was performed once. Although ACT was 250 s, an acute hypermobile thrombus measuring 11 × 5 mm was detected on the left atrial side of the PFO device (see Supplementary material online, *Videos S2* and *S3*; *Figure 1A* and *B*). The patient did not have any symptoms. After consultation with the brain team and exclusion of any acute lesion on brain scan including haemorrhagic transformation, slow t-PA infusion of low dose (25 mg/6 h) was started. The t-PA infusion was started ∼1 h after the thrombus was detected. Due to acute PFO device-related thrombus on the left side, which was mobile and larger than 10 mm, slow infusion of low-dose t-PA treatment was given to avoid any thromboembolic complication. After the administration of a single-dose t-PA, control TEE was performed, which showed the lysis of the thrombus (see Supplementary material online, *Videos S4* and *S5*; *Figure 1C* and *D*). The patient did not have any complications during the treatment. Afterwards, the patient was treated with anticoagulant (warfarin) therapy with a target of international normalized ratio of 2–3 and antiplatelet (clopidogrel) therapy. The reason for choosing warfarin as an initial anticoagulant in the patient was due to the fact that direct initiation of new oral anticoagulants (NOAC) is not allowed in our country associated with local government health insurance plan. The patient was discharged uneventfully, and no complications were detected during follow-up. The patient underwent transthoracic echocardiography and TEE in the first month after the procedure, which did not reveal any PFO device-related thrombus. Warfarin was discontinued after 1 month due to complete lysis of the thrombus. The patient is currently being followed up with clopidogrel.

## Discussion

Patent foramen ovale is a common congenital abnormality and occurs in 20–34% of the population. In most adults, PFO is detected as an incidental finding during cardiac examination. Some PFOs may open widely, allowing thrombus, air, or vasoactive peptides to pass from the venous circulation to the arterial circulation, causing paradoxical embolism. This is associated with clinical events such as cryptogenic paralysis, systemic embolism, migraine with aura, and decompression sickness in divers. Percutaneous PFO closure provides resolution of PFO-related pathologies in carefully selected individuals.^1–3^

Thrombus formation on devices is a recognized postprocedural complication. Patent foramen ovale device thrombosis can occur acutely or during late term. Device thrombosis often develops due to inappropriate anticoagulation or natural coagulation disorders that were not detected before device placement.^4,5^ There is still no definitive evidence about the best follow-up management and antithrombotic treatment regimen after PFO closure.^6^ Until now, surgical and antiplatelet/anticoagulant treatments have been applied to acute thrombosis of PFO device. Vanderheyden *et al*. reported that they treated the PFO device-related thrombus that occurred under optimal anticoagulant treatment with a combination of 100 mg intravenous thrombolytic therapy and glycoprotein IIb/IIIa inhibitors.^7^ In a study authored by Kramer *et al*.,^8^ successful results with surgery, anticoagulant and antiplatelet treatments were achieved in different cases with PFO device-related thrombus. Lezcano *et al*. treated acute PFO device-related thrombosis, which was similar to our case, with 100 mg acetylsalicylic acid, 75 mg clopidogrel, and enoxaparin.^9–11^

Slow infusion of low-dose thrombolytic therapy was first reported by Özkan *et al*. and Aykan AÇ *et al.*,^12–14^ and it was used in the management of mechanical prosthetic valve thrombosis. Studies have reported that slow infusion of low-dose thrombolytic therapy is an effective and well-tolerated strategy. This treatment has also been used in the treatment of peripheral embolism and cerebrovascular events caused by mechanical prosthetic valves. Additionally, slow infusion of low-dose thrombolytic therapy has also become a treatment option in pulmonary embolism and left ventricular assist device-related thrombosis.^15–17^

In the current case with acute development of PFO device-related thrombus, slow infusion of low-dose t-PA, a treatment protocol with previously proven effectiveness and reliability, was considered as the size of the thrombus was larger than 10 mm and hypermobile, the patient had no neurological complaints, and the brain CT scan was free of haemorrhagic transformation. Thus, we were able to successfully lyse the device thrombus in the patient by reducing the risks of bleeding and other potential embolic complications.

In particular, the good results of t-PA treatment of obstructive and non-obstructive thrombus in mechanical prosthetic valves were effective in our decision on t-PA treatment for this patient.

## Conclusion

Performing percutaneous PFO closure under TEE guidance is essential in terms of evaluating the success, results, and complications of the procedure. Acute PFO device-related thrombosis is a rare but important complication. If there is no contraindication for thrombolytic treatment in acutely developed large PFO device thrombosis, slow infusion of low-dose t-PA may be a useful treatment strategy.

## Lead author biography


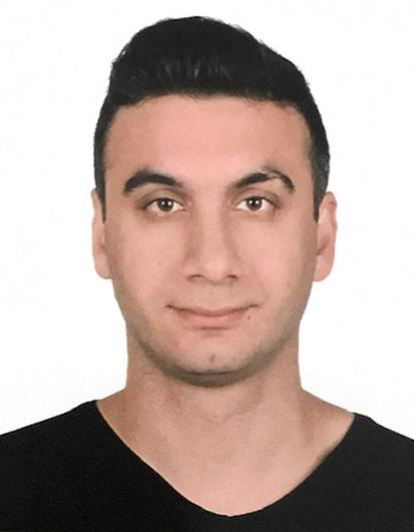


Emrah Bayam works as a cardiologist at Kartal Kosuyolu High Specialty Training and Research Hospital, Department of Cardiology, University of Medical Sciences, Istanbul, Turkey.

## Supplementary Material

ytae360_Supplementary_Data

## Data Availability

The data underlying this article are available in the article and in its online Supplementary material.

## Notes

**Consent:** The authors confirm that written consent for submission and publication of this case report, including images and associated text, has been obtained from the patient in line with COPE guidance.
